# Activation of the Thiazide-Sensitive Sodium-Chloride Cotransporter by Beta3-Adrenoreceptor in the Distal Convoluted Tubule

**DOI:** 10.3389/fphys.2021.695824

**Published:** 2021-08-13

**Authors:** Serena Milano, Monica Carmosino, Andrea Gerbino, Ilenia Saponara, Dominga Lapi, Massimo Dal Monte, Paola Bagnoli, Maria Svelto, Giuseppe Procino

**Affiliations:** ^1^Department of Biosciences, Biotechnologies and Biopharmaceutics, University of Bari, Bari, Italy; ^2^Department of Sciences, University of Basilicata, Potenza, Italy; ^3^Department of Biology, University of Pisa, Pisa, Italy

**Keywords:** beta3-adrenoreceptor, distal convoluted tubule, thiazide-sensitive Na-Cl cotransporter, sympathetic regulation, sodium-chloride cotransporter

## Abstract

We previously showed that the beta-3 adrenergic receptor (BAR3) is expressed in most segments of the nephron where its agonism promotes a potent antidiuretic effect. We localized BAR3 in distal convoluted tubule (DCT) cells expressing the thiazide-sensitive sodium-chloride cotransporter (NCC). Aim of this study is to investigate the possible functional role of BAR3 on NCC modulation in DCT cells. Here, we found that, in mice, the knockout of BAR3 was paralleled by a significant attenuation of NCC phosphorylation, paralleled by reduced expression and activation of STE-20/SPS1-related proline-alanine-rich kinase (SPAK) and WNKs the main kinases involved in NCC activation. Conversely, in BAR1/2 knockout mice, we found reduced NCC abundance with no changes in the phosphorylation state of NCC. Moreover, selective BAR3 agonism promotes both SPAK and NCC activation in wild-type mouse kidney slices. In conclusion, our findings suggest a novel role for BAR3 in the regulation of NCC in DCT.

## Introduction

The beta-adrenergic system regulates numerous renal functions. Three subtypes of the beta-adrenoreceptors (BARs) are known as: BAR1, BAR2, and beta-3 adrenergic receptor (BAR3). While the expression and physiological roles of BAR1 and BAR2 in the kidney are widely documented, evidence regarding the renal expression of BAR3 was lacking until a few years ago. In particular, BAR1 is expressed in the mesangial cells, juxtaglomerular cells, macula densa epithelium, distal tubules, and collecting ducts. BAR2 is expressed mainly in the proximal, distal tubules, and collecting ducts (Boivin et al., 2001; Sata et al., 2018). Stimulation of both BAR1 and BAR2 participate in the regulation of glomerular filtration, sodium reabsorption, acid-base balance, and renin secretion.

We recently demonstrated that BAR3 is expressed in most of the nephron segments also expressing the type-2 vasopressin receptor (AVPR2), including the thick ascending limb (TAL) of Henle, the distal convoluted tubule (DCT), and the cortical and the outer medullary collecting duct (CD; Procino et al., 2016). In particular, we showed in mice that stimulation of BAR3 reduced urine excretion of water, Na^+^, K^+^, and Cl^−^, as a result of increased plasma membrane expression of the water channel aquaporin 2 in the CD and increased the activation of the Na-K-2Cl symporter (NKCC2) in the TAL. Both proteins are key players in the urine concentrating process (Procino et al., 2016).

Here, we focused on the possible functional role of BAR3 in DCT where it localized at the basolateral membrane of epithelial cells expressing the thiazide-sensitive sodium-chloride cotransporter (NCC) at the apical side (Procino et al., 2016; Poulsen et al., 2021). NCC tightly tunes renal sodium reabsorption in DCT to fit blood pressure changes.

NCC activation is a consequence of its phosphorylation by a complex network of kinases including the with-no-lysine kinases WNK1 and WNK4, the STE-20/SPS1-related proline-alanine-rich kinase (SPAK), and Oxidative Stress Response 1 (Pacheco-Alvarez et al., 2006; Richardson et al., 2008; Huang and Cheng, 2015; Hadchouel et al., 2016).

Recent studies showed a direct influence of norepinephrine released by sympathetic nerves on NCC expression and activity (Rojas-Vega and Gamba, 2016). For instance, norepinephrine can increase NCC expression and phosphorylation by activating the BAR2-WNK4 pathway (Mu et al., 2011). Terker et al. (2014) reported that acute stimulation of DCT cells with norepinephrine increased NCC phosphorylation *via* BAR1 activation (Terker et al., 2014). Thus, the aim of the present study is to uncover the possible role of BAR3 stimulation in regulating NCC expression and activation in the DCT cells.

Interestingly, we found that in BAR3 knockout (ko) mice, the amount of phosphorylated NCC (pNCC) was significantly reduced, thus suggesting a regulatory role of BAR3 on NCC. The evidence was confirmed by the observation that BAR3 agonism promoted NCC phosphorylation in vital kidney slices from wt mice but not in those from BAR3 KO animals.

## Materials and Methods

### Antibodies and Reagents

Selective BAR3 agonist BRL37344 (cat. no. sc-200154) and specific BAR3 antagonist L748337 (cat. no. sc-204044) were from Santa Cruz Biotechnology. The PKA inhibitor H-89 (cat. no. B1427) and (deamino-Cys1, D-Arg8)-vasopressin [1-deamino-8-D-argininevasopressin (dDAVP), cat. no. V-1005] were from Sigma (St. Louis, MO).

Antibodies anti-NCC (cat. no. SPC-402D) were from StressMarq Biosciences Inc. (Victoria, BC, Canada). Antibodies against the phosphorylated Thr 53 NCC (cat. no. p1311-53) were from Phosphosolutions. Anti-SPAK (cat. no. S669D) and anti-phospho Ser 373 SPAK (cat. no. S670B), anti-full-length WNK1 (cat. no. S062B), anti-phospho (Ser 382) WNK1 (cat. no. S099B), and anti-WNK4 (cat. no. S064B) antibodies were purchased from MRC-Protein Phosphorylation & Ubiquitylation Unit, University of Dundee, Scotland. Antibodies against PKC (isoform α; cat. no. 2056), phospho-PKC (pan; βII Ser660; cat. no. 9371), ERK1/2 (cat. no. 4695), and phospho-ERK1/2 (Thr202/Tyr204; cat. no. 4370) were from Cell Signaling Technology. Hydrochlorothiazide (HCTZ; cat. no. H4759) was from Sigma-Aldrich.

### Animals

Procedures involving animals were carried out in compliance with the Italian guidelines for animal care (DL 26/14) and the European Communities Council Directive (2010/63/UE). Procedures were approved by the Ethical Committee in Animal Experiments of the University of Bari.

Mice were maintained on a 12-h light/12-h dark cycle, with free access to water and food (2018 Teklad rodent diet, Envigo), in accordance with the Italian Institute of Health Guide for the Care and Use of Laboratory Animals.

Knockout mice, BAR3, and BAR1/2 were derived from two distinct strains characterized by a different genetic background, for this reason, we used specific wild-type mice for each BARs ko model.

BAR3 ko and their wild-type mice (Boss et al., 1999) were purchased from the Jackson Laboratory (Bar Harbor, ME, United States). BAR1/2 ko and wild-type mice (Rohrer et al., 1999) were generated as previously described (Bernstein et al., 2005; Ecker et al., 2006). Experiments were conducted in male mice 4 months old.

Systolic blood pressure was measured in anesthetized mice by means of the “tail-cuff” sphygmomanometer method, as previously described (Martelli et al., 2013).

Metabolic cages were used to collect urine. Mice received a single i.p. injection (200 μl) of HCTZ (50 mg/kg) dissolved in DMSO and diluted 1:10 in sterile saline. Controls received the same volume of vehicle alone. Plasma and urine electrolytes were measured using the ion selective electrode method, aldosterone with R. I. A method.

### Immunofluorescence

Mouse kidneys were fixed overnight with 4% paraformaldehyde at 4°C, cryopreserved in 30% sucrose for 24 h, and then embedded in optimal cutting temperature medium. Thin cryosections (7 μm) were subjected to immunofluorescence analysis as previously reported (Procino et al., 2016). Sections were incubated with the primary antibodies anti-NCC, anti-pNCC, anti-SPAK, anti-pSPAK, and the appropriate AlexaFluor-conjugated secondary Ab (Life Technologies) according to the manufacturer’s instructions. Confocal images were obtained with a confocal laser-scanning fluorescence microscope (Leica TSC-SP2, Mannheim, Germany). For the quantification of the fluorescence intensity (FI expressed as arbitrary units), 18 confocal images (three for each mouse) were analyzed blindly for each genotype using ImageJ software. Images were then background subtracted and the appropriate threshold was automatically calculated for the two separate channels (green and red) obtained from each image. Then, the mean fluorescence intensity of each image was quantified using ImageJ.

### Immunoblotting

Whole kidney isolated from wild type, BAR3 and BAR1/2 ko mice were homogenized in RIPA buffer as previously described (Procino et al., 2014). 30 μg of each lysate was separated by SDS-PAGE using Mini-PROTEAN^®^ TGX Stain-Free^™^ Precast Gels Bio-Rad and analyzed by Western blotting as previously described (Milano et al., 2018). Densitometry was performed using the Image Lab^™^ software (Bio-Rad) of ChemiDoc^™^ (Bio-Rad) imaging system, after normalization for the total protein content using the Stain-Free^™^ technology (Bio-Rad) according to manufacturer’s instructions.

### Kidney Slices

Sex-, age-, and weight-matched C57BL/6J (*N* = 6) or BAR3 ko (*N* = 6) male mice were used for the kidney slices experiments. Mice were anesthetized with tribromoethanol (250 mg/kg) and killed by cervical dislocation. Kidneys were collected and thin transversal slices (250 μm) were obtained using a Mcllwain Tissue Chopper (Ted Pella Inc.; Redding, CA, United States). Slices were left at 37°C in Dulbecco’s Modified Eagle Medium/F12 medium (CTR) or stimulated for 40 min with dDAVP (10^–7^ M) or BRL37344 (10^–5^ M), the latter given alone or after 30 min of preincubation with either L748,337 (10^–7^ M) or H89 (10^–5^ M). Slices were processed for Western blotting experiments as described above or fixed in 4% paraformaldehyde and thin cryosections (7 μm) were subjected to immunofluorescence. For the quantification of fluorescence intensity, 30 confocal images (six for each condition) were analyzed.

### Statistical Analysis

For statistical analysis, GraphPad Prism software (La Jolla, CA) was used. Unpaired Student’s *t*-test was used to compare knockout and wild-type mice of each strain. For multiple comparison, one-way ANOVA was performed. All values are expressed as means ± SEMs. A difference of *p* < 0.05 was considered statistically significant. Details about statistical analyses are reported in figure legends.

## Results

### Blood Chemistry of Animals Used in the Study

We previously characterized blood chemistry of BAR3 ko mice (Procino et al., 2016). Here, we compared blood chemistry between BAR3 ko, BAR1/2 ko mice, and their wt controls. As reported in Table 1, plasma Na^+^, K^+^, and Cl^−^ did not change significantly between genotypes. Plasma aldosterone concentrations were not significantly altered in BARs knockout, although they were higher in the genetic background in which BAR1/2 ko mice were generated (C56BL6), compared to FVB animals in which BAR3 ko mice were obtained. Systolic blood pressure was measured in all mouse genotypes and did not show significant differences in agreement with previously published data (Rohrer et al., 1999; Deschepper et al., 2004; Moens et al., 2009).

**Table 1:** Plasma electrolytes, aldosteron and blood pressure in each genotype analyzed in the study.

Parameter (plasma)	BAR3 wt	BAR3 KO	BAR1/2 wt	BAR1/2 KO
Na^+^ (mEq/L)	138 ± 3.05	141.3 ± 2.40	140 ± 1.15	139.3 ± 2.9
K^+^ (mEq/L)	6.17 ± 1.6	5.77 ± 0.49	5.87 ± 0.39	6.63 ± 1.24
Cl^−^ (mEq/L)	102.7 ± 0.67	104 ± 2	101.3 ± 1.76	100 ± 1.15
Aldosterone (pg/ml)	204.7 ± 14.62	172.7 ± 16.18	318.7 ± 16.18	333.3 ± 24.04
Blood pressure (mmHg)	117 ± 1.8	120 ± 1.4	119 ± 2.1	119.6 ± 2

Values are means ± SEM of measurements in six mice/genotype. Statistical analysis was performed using an unpaired *t*-test.

### BAR3 Ko Mice Show Reduced Urine Na^+^ Excretion Upon HCTZ Administration

**Figure 1:**
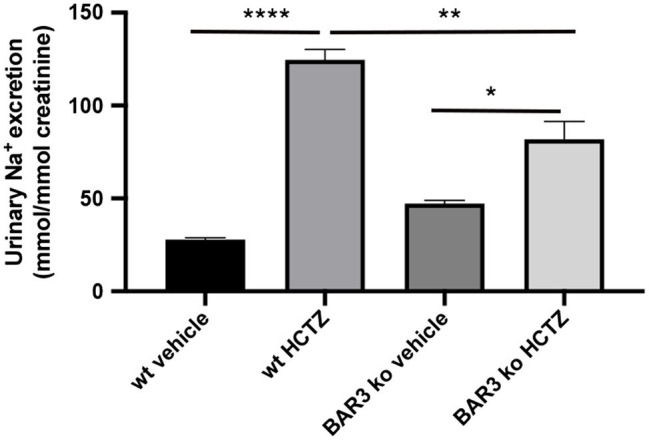
Effect of hydrochlorothiazide diuretic on natriuresis in BAR2 ko mice. BAR3 ko mice and their age-matched wt controls (*N* = 6 for each group) were individually housed in metabolic cages for 24 h. Three animals for each group received an intraperitoneal injection of 50 mg/Kg HCTZ in sterile saline, while control animals received the vehicle alone. Urines were collected for 6 h after injection and Na^+^ excretion analyzed and reported as mean values ± SEM. Both genotypes significantly increased natriuresis in response to HCTZ, although in BAR3 ko mice, this effect was significantly blunted compared to wt animals. Statistical analysis was performed by one-way ANOVA followed by Bonferroni multiple comparison test. ^*^p < 0.05; ^**^p < 0.01; and ^****^p < 0.0001.

Although BAR3 ko mice did not show defects in plasma Na^+^ handling, to test the hypothesis that gene deletion of the BAR3 receptor might affect the regulation of the NCC transporter in the DCT, we analyzed the urinary excretion of Na^+^ after the administration of the diuretic HCTZ, a specific inhibitor of NCC. BAR3 ko mice and their wt controls (six for each genotype) were housed and acclimatized for 24 h in metabolic cages, i.p. injected with HCTZ (50 mg/Kg) or vehicle alone, and urine collected for the next 6 h post-injection. Increase of urine output confirmed the effect of the diuretic (not shown). Urine Na^+^ excretion normalized for creatinine is reported in Figure 1. Among vehicle-injected animals, BAR3 ko mice had an increased Na^+^ excretion compared to wt animals, as we previously reported (Procino et al., 2016). Interestingly, although HCTZ induced natriuresis in both genotypes, BAR3 ko mice showed a significantly reduced natriuresis compared to their wt controls, likely suggesting that BAR3 ko might have less active NCC to inhibit.

### BAR3 Knockout Mice Show Reduced NCC Phosphorylation and Reduced Levels of SPAK and pSPAK

**Figure 2:**
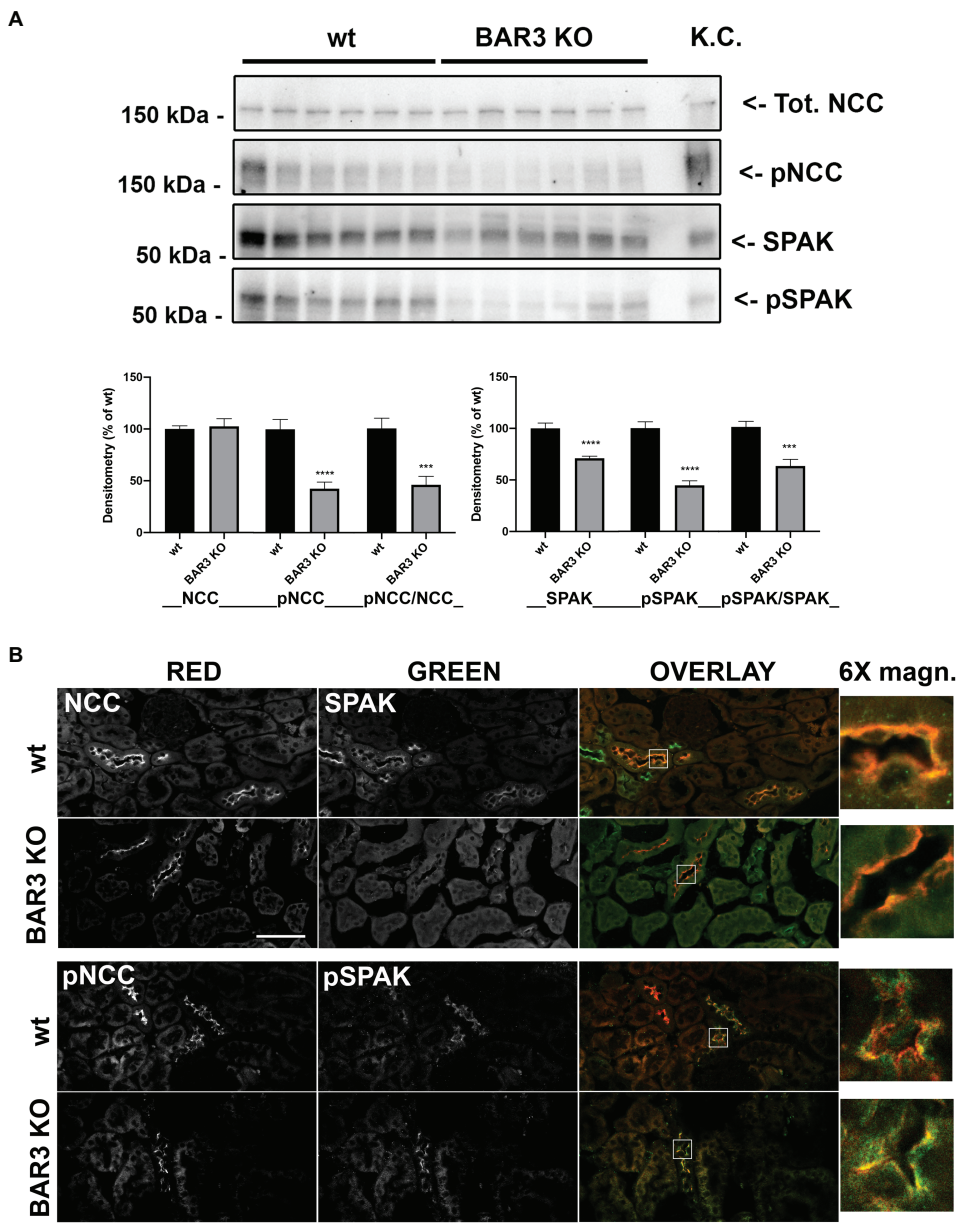
Effect of BAR3 knockout on total and phosphorylated NCC (pNCC) and SPAK in the kidney. **(A)** Western blotting analysis of total and phosphorylated forms of both NCC and SPAK on total kidney homogenates from both wt and BAR3 ko mice. Protein extract from mouse kidney cortex (KC) was loaded as positive control. Densitometric analysis of NCC, pNCC, SPAK, and pSPAK bands, normalized to total lane protein content using the Stain-Free^™^ gels technology, from three independent experiments was reported as percentage of wt mice. Data are provided as mean ± SEM. ^***^p < 0.001; ^****^p < 0.0001 assessed by Student’s *t*-test. *N* = 6 per group. **(B)** Kidney sections of wild type (wt) and BAR3 ko mice were subjected to immunofluorescence co-localization of NCC and SPAK or pNCC and pSPAK. Representative images are shown. *N* = 6 per group (bar = 25 μm).

Based on the reduced effect of HCTZ in BAR3 ko mice, we investigated the abundance and activation of NCC in these animals. To this end, kidneys from wild type and BAR3 ko mice were subjected to Western blotting and immunofluorescence experiments to assess the abundance and localization of both total and pNCC. Western blotting analysis of total kidney lysates showed no difference in the abundance of total NCC between wt and BAR3 ko mice (Figure 2A). Conversely, pNCC was significantly reduced of about 60% in the kidney of BAR3 ko mice (Figure 2A) also after normalization to total NCC expression. It is known that SPAK activity is involved in NCC phosphorylation/activation. Therefore, we investigated whether the levels of total SPAK and its phosphorylated/activated form, pSPAK, were also changed in BAR3 ko animals. Western blotting analysis of kidneys lysates revealed that in BAR3 ko, compared to wt mice, both total and pSPAK were reduced by 30 and 60%, respectively (Figure 2A). After normalization to total SPAK, pSPAK was reduced by 40%. Immunofluorescence analysis of contralateral kidneys showed that NCC immunoreactivity in both genotypes was clearly concentrated at the apical plasma membrane of DCT cells (Figure 2B 6× magnification inset). The fluorescence intensity (FI) and subcellular distribution of NCC staining was comparable between BAR3 ko and wt mice (NCC FI: wt 5304 ± 95 vs. BAR3 ko 5597 ± 180). On the other hand, the intensity of pNCC staining in kidney sections from BAR3 ko mice was reduced compared to wt (pNCC FI: wt 10824 ± 350 vs. BAR3 ko 3894 ± 120, *p* < 0.0001) (Figure 2B).

As for total and pSPAK, confocal microscopy analysis supported the results obtained by Western blotting experiments. Figure 2B shows that both total and pSPAK were consistently less abundant in DCTs of BAR3 ko compared to wt mice (SPAK FI: wt 4067 ± 150 vs. BAR3 ko 2357 ± 130, *p* < 0.0001; pSPAK FI: wt 7105 ± 550 vs. BAR3 ko 3560 ± 370, *p =* 0.0003).

Together, the data suggest that BAR3 might be involved in the regulation of NCC phosphorylation/activation and the lack of BAR3 impaired the abundance and activation of SPAK.

### Knockout of BAR1/2 Reduces NCC Expression Level but Does Not Affect Abundance and Activation of SPAK

**Figure 3:**
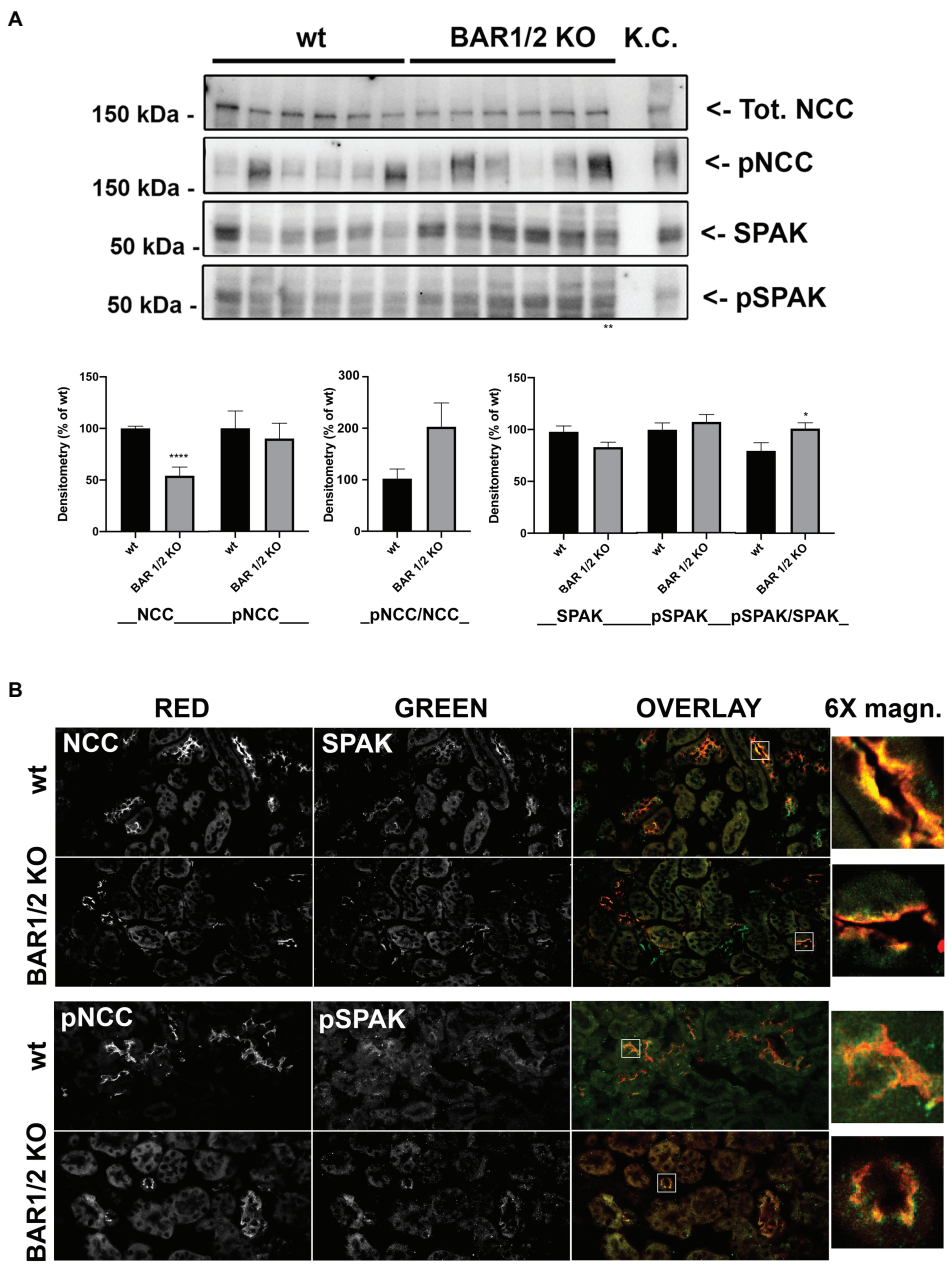
Effect of BAR1/2 knockout on total and pNCC and SPAK in the kidney. **(A)** Western blotting analysis of total and phosphorylated forms of both NCC and SPAK on total kidney homogenates from both wt and BAR1/2 ko mice. Protein extract from mouse kidney cortex (KC) was loaded as positive control. Densitometric analysis of NCC, pNCC, SPAK, and pSPAK bands, normalized to total lane protein content using the Stain-Free^™^ gels technology, from three independent experiments was reported as percentage of wt mice. Data are provided as mean ± SEM. ^*^p < 0.05; ^****^p < 0.0001 assessed by Student’s *t*-test. *N* = 6 per group. **(B)** Kidney sections of wild type (wt) and BAR1/2 ko mice were subjected to immunofluorescence co-localization of NCC and SPAK or pNCC and pSPAK. Representative images are shown. *N* = 6 per group (bar = 25 μm).

To dissect the contribution of BAR subtypes in the sympathetic regulation of NCC, we analyzed the effects of the double knockout of BAR1 and BAR2 on NCC expression and activation, performing the same analysis reported above for BAR3 ko mice. As showed in Figure 3A, total NCC was reduced by 40% in BAR1/2 ko mice compared to their wt. In contrast, despite the high variability of pNCC levels between mice, the overall variation of pNCC between BAR1/2 ko and wt animals was not statistically significant (Figure 3A). After normalization to total NCC, pNCC showed an increase that was not statistically significant.

We also evaluated the levels of SPAK e pSPAK in BAR1/2 ko and their wild-type mice and we found no significant difference. After normalization to total SPAK, pSPAK was significantly increased in BAR1/2 ko mice.

These results were supported by immunofluorescence experiments showed in Figure 3B: The lack of BAR1/2 reduced the intensity of NCC staining in kidney sections (NCC FI: wt 4051 ± 210 vs. BAR 1/2 ko 1526 ± 186, *p* < 0.0001). Conversely, pNCC, although highly variable, did not change compared to wt mice (pNCC FI: wt 4869 ± 760 vs. BAR 1/2 ko 4580 ± 950, *p* = 0.8168; Figure 3B).

In addition, neither the abundance nor the phosphorylation state of SPAK changed in immunofluorescence experiments performed on BAR1/2 knockout mice (SPAK FI: wt 1325 ± 246 vs. BAR 1/2 ko 1238 ± 215, *p* = 0.7961; pSPAK FI: wt 1492 ± 118 vs. BAR 1/2 ko 1517 ± 236, *p* = 0.9266). These results support the hypothesis that BAR1 and BAR2 might participate in the control of NCC abundance rather than the phosphorylation/activation of the cotransporter.

### Involvement of Upstream Regulators of NCC Activation

**Figure 4:**
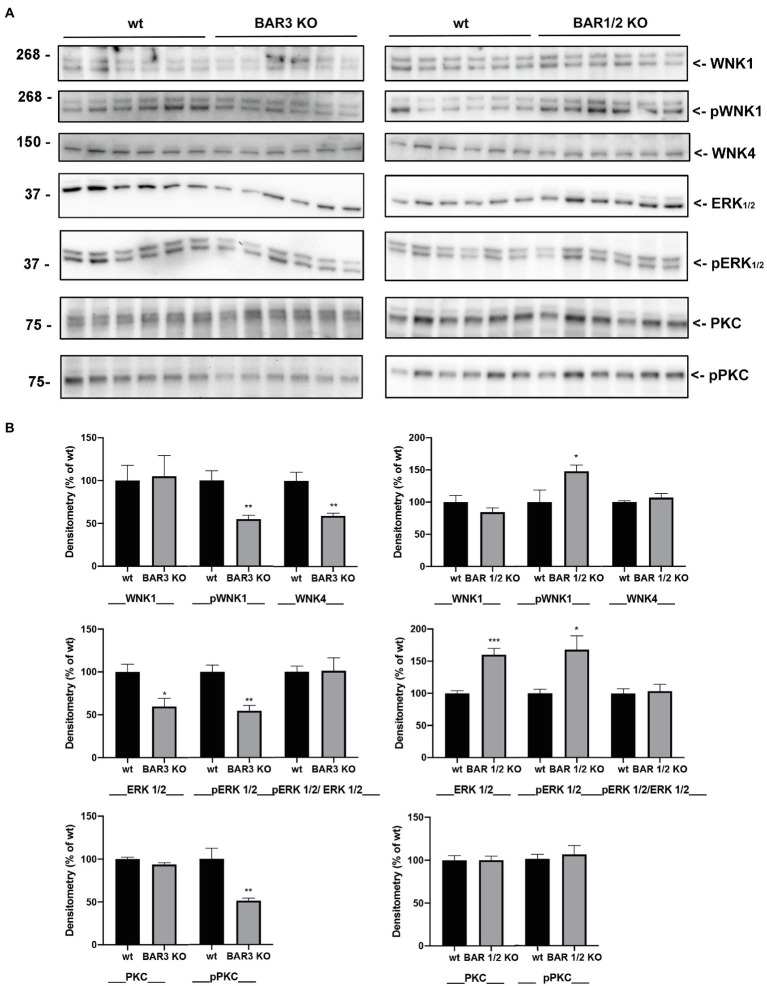
**(A)** Western blotting analysis of total and phosphorylated (p) forms of WNK1, ERK1/2, and PKC and total WNK4, on total kidney homogenates from both BAR3 ko and BAR1/2 ko mice, compared to their respective wt mouse strains. Molecular weight in kDa is indicated on the left of each lane. **(B)** Densitometric analysis of each protein bands, normalized to total lane protein content using the Stain-Free^™^ gels technology, from three independent experiments was reported as percentage of wt mice. Data are provided as mean ± SEM. ^*^p < 0.05; ^**^p < 0.01; and ^***^p < 0.001 assessed by Student’s *t*-test. *N* = 6 per group.

We further investigated the possible effect of BARs knockdown on upstream regulators of SPAK activity and/or on the activity of PKC and ERK1/2. As reported in Figure 4, in total kidney lysates from BAR3 ko animals, pWNK, but not the total levels of WNK1, was significantly reduced compared to the wt strain. Also, the expression of total WNK4 showed a significant reduction. On the other hand, when we analyzed the same kinases in kidneys from BAR1/2 ko mice, we found a significant increase of active WNK1.

As for ERK1/2, we found that total ERK1/2 was reduced in BAR3 ko mice, compared to their controls and this reflected on a parallel reduction of phosphorylated ERK1/2. Overall, after normalization of each form for the total protein load, the ratio between pERK/ERK was unchanged between BAR3 ko and their wt controls, likely suggesting that of ERK1/2 signaling is not affected by BAR3 ko. In kidneys from BAR1/2 ko animals, the upregulation of total ERK1/2 abundance was, as expected, paralleled by a significant increase of pERK1/2, but again, no changes in the ratio of pERK/ERK suggested that the also absence of functional BAR1/2 did not perturb ERK1/2 signaling. The effect of BARs ko on the PKC signaling pathway was investigated with an antibody against total PKC (*α* isoform) and a pan-antibody against phosphorylated PKC isoforms (pPKC). As shown in Figure 4, regardless of the genotype investigated, the total expression of PKC did not change, but a significant reduction of active pPKC was observed only in BAR3 ko mice, compared to their wt strain.

### BAR3 Stimulation Promotes NCC Activation in Kidney Slices

Kidney slices from wt mice were incubated with the selective BAR3 agonist BRL37344, alone or after treatment with either L-748,337 (a specific BAR3 antagonist) or H89 (a PKA inhibitor). Stimulation with dDAVP was used as control for NCC and SPAK phosphorylation.

**Figure 5:**
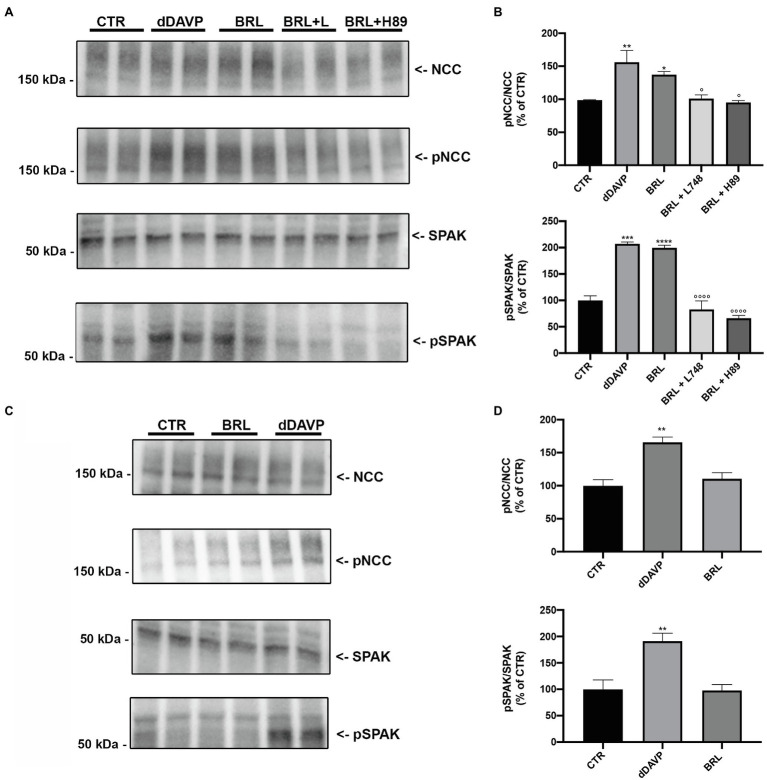
Semi-quantitative analysis of the abundance of both pNCC and pSPAK after BAR3 stimulation in kidney slices. **(A,B)** Freshly isolated kidney slices from 6 wt mice were left untreated (CTR) or incubated for 40 min with dDAVP (10^–7^ M) or BRL37344 (BRL, 10^–5^ M), the latter given alone or after 30 min of preincubation with either the BAR3 antagonist L748337 (L748 10^–7^ M) or the protein kinase A inhibitor H89 (10^–5^ M). Representative immunoblots showed the effect of these treatments on the total and phosphorylated forms of NCC and SPAK. Each lane represents a lysate of kidney slices from three wt mice. The densitometric analysis, expressed as percentage of the CTR, from three independent experiments was reported. Error bars represent the SEM. ^*^Indicates the comparison vs. CTR, °indicates the comparison vs. BRL; ^*^, ^°^*p* < 0.05; ^**^p < 0.01; ^***^p < 0.001; ^****^, ^°°°°^*p* < 0.0001 assessed by one-way ANOVA followed by Dunnett’s multiple comparison test. **(C,D)** Freshly isolated kidney slices from 6 BAR3 ko mice were stimulated with BRL and dDAVP as described above. Each lane represents a lysate of kidney slices from three mice. The densitometric analysis, expressed as percentage of the CTR, from three independent experiments was reported. Error bars represent the SEM. ^**^p < 0.01 vs. CTR assessed by one-way ANOVA followed by Dunnett’s multiple comparison test.

Figures 5A,B reported the Western blotting semi-quantitative analysis of the abundance of both pNCC and pSPAK, normalized for the total content of NCC and SPAK, respectively. Normalization of the phosphorylated form of both proteins to their total content was necessary considering that in kidney slices the number of DCT may be heterogeneous. Incubation with BRL37344 increased the phosphorylation of NCC and SPAK (of about 40 and 200%, respectively) and this effect was significantly prevented by both L748337 and H89. In kidney slices from BAR3 ko mice (Figures 5C,D), as expected, BRL 37344 did not increase the phosphorylation of NCC and SPAK, thus confirming that the effect of BRL 37344 observed in wt animals was exclusively due to the BAR3 activation. A parallel set of kidney slices, treated as described above, was used for immunofluorescence analysis.

**Figure 6:**
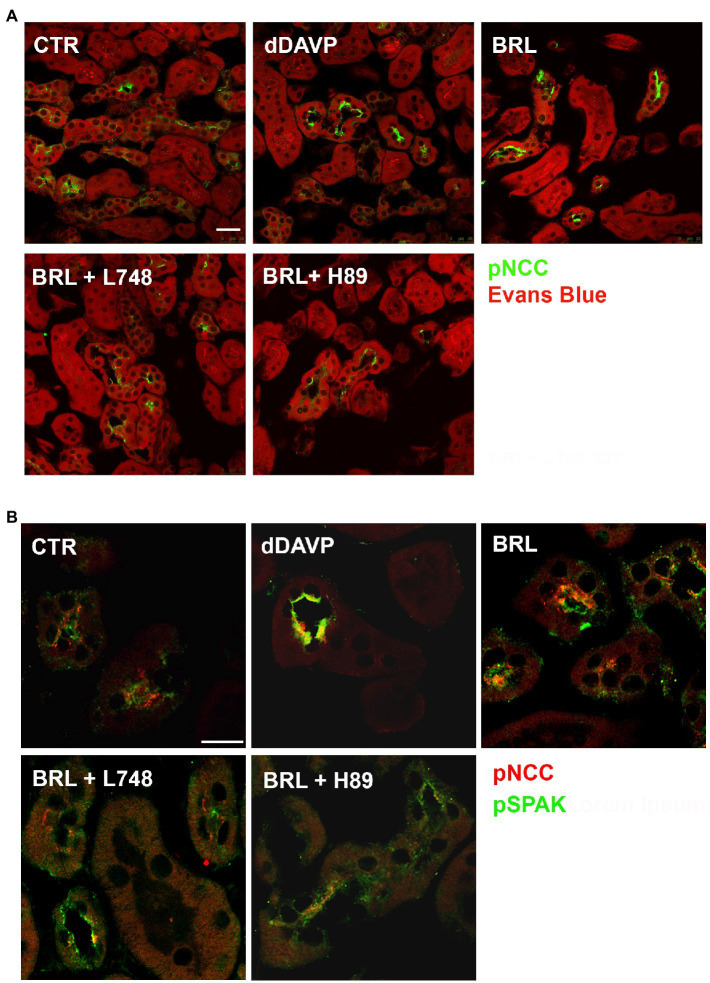
In kidney slices, selective BAR3 stimulation promotes NCC phosphorylation by activating PKA and SPAK in DCT cells. Freshly isolated kidney slices from 6 wt mice were incubated for 40 min at 37°C in culture medium alone (CTR) or with dDAVP (10^–7^ M) or BRL37344 (BRL, 10^–5^ M), the latter given alone or after 30 min of preincubation with either the BAR3 antagonist L748,337 (L748 10^–7^ M) or the protein kinase A inhibitor H89 (10^–5^ M). **(A)** Immunofluorescence images of kidney sections showing pNCC (green) and counterstain with Evans blue (red; bar = 25 μm). **(B)** Confocal microscopy showed that the treatment with BRL increased the abundance of both pNCC and pSPAK at the apical plasma membrane of DCT cells. The BRL effect was prevented by either L748 or H89 (bar = 25 μm).

Confocal microscopy reported in Figure 6A showed that both BRL37344 and dDAVP increased the pNCC fluorescence signal, which was clearly brighter compared to that observed in resting slices (pNCC FI: CTR 3043 ± 180 vs. BRL 5560 ± 250, *p* < 0.0001; CTR vs. dDAVP 6485 ± 195, *p* < 0.0001). L748,337 prevented the effect of BRL37344 demonstrating that the increase in NCC phosphorylation was specifically attributable to BAR3 stimulation (pNCC FI: BRL + L748 2328 ± 210 vs. CTR ns – vs. BRL *p* < 0.0001). H89 prevented the effect of BRL37344 indicating the downstream involvement of PKA in the phosphorylation of NCC (pNCC FI: BRL + H89 2486 ± 168, vs. CTR ns – vs. BRL *p* < 0.0001).

Next, we also evaluated the levels of SPAK phosphorylation under the same experimental conditions. In Figure 6B, immunofluorescence analysis showed that the increase of pNCC induced by BRL37344 was paralleled by a concomitant increase of pSPAK (pSPAK FI: CTR 2299 ± 250 vs. BRL 5863 ± 250, *p* < 0.0001; CTR vs. dDAVP 7024 ± 230, *p* < 0.0001). Pretreatment with either L748,337 or H89 prevented the effect of BRL37344 on the phosphorylation of both NCC and SPAK (pSPAK: BRL + L748 1929 ± 278 vs. CTR ns – vs. BRL *p* < 0.0001; BRL + H89 2997 ± 300, vs. CTR ns – vs. BRL *p* < 0.0001).

## Discussion

We previously showed the expression of BAR3 in the same nephron segments expressing AVPR2 and its role in promoting water and salt reabsorption (Procino et al., 2016). Interestingly, BAR3 ko mice exhibit an increased Na^+^, K^+^, and Cl^−^ urine excretion (Procino et al., 2016), a finding that could not be explained only by the reduced activation of NKCC2 that we demonstrated in these animals. In fact, the main finding of the present study is that BAR3 regulates the activation of NCC in DCT cells.

Three evidence clearly support this conclusion: (1) HCTZ shows a reduced natriuretic effect *in vivo* in BAR3 ko mice; (2) BAR3 ko mice exhibit reduced pNCC levels compared to their controls; and (3) in vital mouse kidney slices, the pharmacologic agonism of BAR3 clearly promotes NCC phosphorylation which is prevented by specific BAR3 antagonism.

To date, the study of the relationship between sympathetic stimulation and increased NCC activity has been restricted to the contribution of BAR1 and BAR2. Using BAR1 ko and BAR2 ko mice, Mu et al. demonstrated that norepinephrine increased NCC expression and phosphorylation through the activation of BAR2 (Mu et al., 2011). On the other hand, Terker et al. showed that acute adrenergic stimulation increased the abundance of pNCC *via* BAR1 stimulation in mice (Terker et al., 2014). More recently, it has been shown that pharmacological stimulation of BAR2 with salbutamol increases both NCC phosphorylation and systolic blood pressure, the latter effect blunted by thiazide (Poulsen et al., 2021). In the present study, we demonstrated that also BAR3 plays an additional, crucial role in NCC modulation. Thus, all three subtypes of BARs are implicated in the control of sodium reabsorption by NCC in DCT.

Boivin et al. previously immunolocalized BAR1 and BAR2 at the apical membrane of DCT cells (Boivin et al., 2001), while we found BAR3 at the basolateral membrane of the same cells (Procino et al., 2016). Although the expression of all BARs in the same cell type could appear redundant, their polarized expression in different plasma membrane domains of the cell could suggest their sequential activation (in time and space) that might finely modulate the activation and expression of NCC. It is conceivable that, in chronological order, circulating catecholamines, upon release by sympathetic nerve terminals, might bind basolateral BAR3 and rapidly activate NCC. Upon prolonged release of catecholamines, they might be filtered by the glomerulus, activate apical BAR1 and BAR2 (Boivin et al., 2001), thus also increasing the expression of NCC. This hypothesis is supported by our findings in mice, that the absence of BAR3 decreased the phosphorylation of NCC and the absence of BAR1/2, preserved the levels of NCC phosphorylation likely because BAR3 might be tonically stimulated by catecholamines released by sympathetic nerves.

A key pathway that modulates NCC phosphorylation involves WNK kinases that interacting with SPAK and OSR1 can, in turn, directly phosphorylate the cotransporter (Richardson and Alessi, 2008). Here, we show that, in BAR3 ko, mice pSPAK was markedly reduced, even though total SPAK was also significantly downregulated, albeit to a lesser extent. Accordingly, we found that BAR3 ko mice have reduced levels of upstream SPAK modulators in the kidney, such as phosphorylated WNK1 and WNK4. This observation nicely fits with the current working model seeing WNKs phosphorylating SPAK (Vitari et al., 2005; Richardson and Alessi, 2008; Richardson et al., 2008), which in turn activates NCC by phosphorylation in DCT (Moriguchi et al., 2005; San-Cristobal et al., 2009). Our hypothesis is indeed that the reduction of WNK-SPAK signaling in the kidney upon BAR3 ko induces the reduction of NCC phosphorylation and activity in these mice. Although BAR2 is also involved in the activation of SPAK and WNK (Poulsen et al., 2021), in the kidney of BAR1/2 ko mice, the activity of SPAK and WNK resulted almost normal or slightly upregulated compared to their controls, thus suggesting that BAR3 alone is sufficient to preserve the WNK-SPAK pathway activation in the kidney under sympathetic stimulation.

Further studies are required to elucidate the mechanisms underlying SPAK downregulation in BAR3 ko mice.

We also explored in BAR3 ko mice, the activity of other pathways, either upstream or downstream the WNK-SPAK axis. It has been reported that WNK4 may affect the ERK1/2 pathway, which may in turn influence NCC activation (Zhou et al., 2012). However, we found that the ERK1/2-pERK1/2 ratio was unchanged in BAR3 ko animals compared to their wt controls, likely suggesting that the ERK signaling is not involved in the inactivation of NCC observed in these animals. PKC is also considered a modulator of WNK4 (Shibata et al., 2014), as its activation in physiological conditions phosphorylates Kelch-like 3 motifs of the ubiquitin ligases containing Cullin 3, preventing its binding to WNK4 and reducing its degradation. Interestingly, in our BAR3 ko mouse model, the levels of phosphorylated/active PKC were significantly reduced, in the absence of apparent PKC downregulation. This might explain the reduced expression of WNK4 in these mice. In this scenario, the absence of BAR3 expression at the basolateral membrane should reduce, at the same time, the activation of both PKA, through the Gαs subunit and PKC activation *via* the βγ dimer (Zhong et al., 2001).

The role of the cAMP/PKA pathway triggered by vasopressin in the DCT (Mutig et al., 2010; Pedersen et al., 2010; Saritas et al., 2013) has been recently further enriched by the observations that PKA might also phosphorylate WNK4 and, in turn, mediate the PPAK/OSR1-NCC activation (Castañeda-Bueno et al., 2017). As we demonstrated in renal cells (Milano et al., 2018), BAR3, like the vasopressin receptor AVPR2, is coupled to the cAMP pathway and this would explain the reduced NCC phosphorylation in BAR3 ko mice. Furthermore, the observation reported here, that H89 prevents the effect of BAR3 stimulation on NCC phosphorylation in kidney slices, supports a key role of PKA in the regulation of NCC activity by BAR3.

A limitation of our study could be that we only used male mice. Unfortunately, studies on NCC activation are not suitable in female since it has been reported that NCC expression and activity is higher in female than male due to ovarian hormones and prolactin (Rojas-Vega et al., 2015; Veiras et al., 2017). However, we think that this did not affect the impact of our findings in the scenario of kidney physiology.

This piece of information about the role of BAR3 in regulating NCC function in the DCT has a key importance in the context of the autonomic regulation of water and salt homeostasis in the kidney and, consequently, in the control of blood pressure (Crowley and Coffman, 2014).

It is known that the kidney is densely innervated by sympathetic nerves which activity is increased in hypertension (Sata et al., 2018), as demonstrated by catheter-based renal sympathetic denervation experiments (Krum et al., 2009; Schlaich et al., 2009). The critical role of NCC in the maintenance of blood pressure is also highlighted by genetic diseases as Gitelman (Riveira-Munoz et al., 2007) and Gordon’s syndrome (Simonetti et al., 2012) and by its role in the development of salt-sensitive hypertension (Moes et al., 2014). Matayoshi e al. reported that a BAR3 gene polymorphism (T727C) was susceptible to the antihypertensive effect of thiazide in patients with essential hypertension (Matayoshi et al., 2004).

Our findings unveil a new BAR3-dependent regulatory mechanism of NCC activity in the DCT, which, in addition to the thick ascending limb, the connecting tubule and the collecting duct, play a pivotal role in the fine-tuning of sodium and water excretion. As the role of BAR3, as regulator of multiple segments of the kidney tubule becomes clearer, the emerging frame is that sympathetic activation might sustain stress responses by rapidly increasing blood pressure and blood perfusion in physiological conditions, independently of aldosteron and K^+^ blood levels. The other side of the coin, which needs more in-depth studies, is that the pharmacological antagonism of BAR3 might represent a tool to take hypertension under control.

## Data Availability Statement

The raw data supporting the conclusions of this article will be made available by the authors, without undue reservation.

## Ethics Statement

The animal study was reviewed and approved by the Procedures were approved by the Ethical Committee in Animal Experiments of the University of Bari.

## Author Contributions

SM and GP: conceptualization. SM: methodology and writing – original draft preparation. IS, AG, and MC: formal analysis. IS, SM, and DL: investigation. MD and PB: resources. GP, MD, PB, and MC: writing – review and editing. GP: visualization and supervision. MS: project administration. MS and GP: funding acquisition. All authors have read and agreed to the published version of the manuscript.

## Conflict of Interest

The authors declare that the research was conducted in the absence of any commercial or financial relationships that could be construed as a potential conflict of interest.

## Publisher’s Note

All claims expressed in this article are solely those of the authors and do not necessarily represent those of their affiliated organizations, or those of the publisher, the editors and the reviewers. Any product that may be evaluated in this article, or claim that may be made by its manufacturer, is not guaranteed or endorsed by the publisher.
